# Transparent decision support for mechanical ventilation using visualization of clinical preferences

**DOI:** 10.1186/s12938-021-00974-5

**Published:** 2022-01-24

**Authors:** Stephen Edward Rees, Savino Spadaro, Francesca Dalla Corte, Nilanjan Dey, Jakob Bredal Brohus, Gaetano Scaramuzzo, David Lodahl, Robert Ravnholt Winding, Carlo Alberto Volta, Dan Stieper Karbing

**Affiliations:** 1 Respiratory and Critical Care Group, Department of Health Science and Technology, Aalborg University, Aalborg, Denmark; 2 Department of Morphology, Experimental Medicine and Surgery, Section of Anaesthesia and Intensive Care, Arcispedale Sant’ Anna, University of Ferrara, Ferrara, Italy; 3 Department of Anaesthesia and Intensive Care, Humanitas Clinical and Research Center-IRCCS, Rozzano, MI Italy; 4 Department of Anesthesia and Intensive Care, Regions Hospital Herning, Herning, Denmark; 5 Mermaid Care A/S, Nørresundby, Denmark; 6 Department of Anesthesia and Intensive Care, Aalborg University Hospital, Aalborg University, Aalborg, Denmark

**Keywords:** Mechanical ventilation, Clinical decision support, Clinical preferences

## Abstract

**Background:**

Systems aiding in selecting the correct settings for mechanical ventilation should visualize patient information at an appropriate level of complexity, so as to reduce information overload and to make reasoning behind advice transparent. Metaphor graphics have been applied to this effect, but these have largely been used to display diagnostic and physiologic information, rather than the clinical decision at hand. This paper describes how the conflicting goals of mechanical ventilation can be visualized and applied in making decisions. Data from previous studies are analyzed to assess whether visual patterns exist which may be of use to the clinical decision maker.

**Materials and methods:**

The structure and screen visualizations of a commercial clinical decision support system (CDSS) are described, including the visualization of the conflicting goals of mechanical ventilation represented as a hexagon. Retrospective analysis is performed on 95 patients from 2 previous clinical studies applying the CDSS, to identify repeated patterns of hexagon symbols.

**Results:**

Visual patterns were identified describing optimal ventilation, over and under ventilation and pressure support, and over oxygenation, with these patterns identified for both control and support modes of mechanical ventilation. Numerous clinical examples are presented for these patterns illustrating their potential interpretation at the bedside.

**Conclusions:**

Visual patterns can be identified which describe the trade-offs required in mechanical ventilation. These may have potential to reduce information overload and help in simple and rapid identification of sub-optimal settings.

**Supplementary Information:**

The online version contains supplementary material available at 10.1186/s12938-021-00974-5.

## Background

Selecting the appropriate mechanical ventilation for patients residing at the intensive care unit (ICU) is a difficult task. Inappropriate settings have been correlated with mortality [1], and the presence of guidelines to modify clinical practice are not always effective [2]. Selecting the appropriate settings for mechanical ventilation appears then to be an area where computer-based clinical decision support systems (CDSS) could be beneficial.

This potential has resulted in the development of CDSS [3–15]. These systems are based on differing technologies for generating advice, including production rules [3–7], or physiological mathematical models [8–13] and artificial neural networks [14, 15] combined with production rules, or utility functions. In addition, these systems function either as closed loop systems directly controlling the ventilator [5–10], or open loop providing advice to the clinician who then controls the ventilator [3, 4, 11–15].

The technological complexity of such systems is, however, not limited to the approach used to determining appropriate settings. Of significant importance is the presentation of information to the user. Indeed, this might be of particular importance in the case of open loop systems, which require a greater level of interaction between the user and system. It is well known that monitoring the patient’s state in the ICU is characterized by information overload [16, 17], and that simple line graph type illustrations are inadequate to effectively present patient state, and therefore, support decisions [18]. Cole proposed the use of metaphor graphics [18], such that the area of rectangles illustrated the status and progression of measured respiratory variables such as depth and frequency of breathing. Horn et al. further developed this approach [19], using metaphor graphical objects including combination of multiple shapes and color into a single object. In this way, they extended the dimensionality of Cole’s approach allowing for representation of measured variables relating to circulation, respiration and fluid balance.

Metaphor graphics may then address the problem of ‘how’ to represent information, but it does not explicitly address the issue as to which information to present and for what purpose. As noted by Seiver and Holtzmann [16], when understanding decisions in the ICU, we should be precise in our use of terms relating to the decision-making process. To understand the patient’s condition, requires presentation of information suitable for diagnosis. To understand decisions, requires representation of information related to the preferences of the decision maker. The difference between these can be illustrated by an example. The presence of acute lung injury may result in poor lung mechanics and gas exchange, and monitoring the presence of these is important in understanding the patient and monitoring their disease progression. In contrast, selecting the correct level of inspired oxygen is a balance between the risks of over oxygenation, e.g. oxygen toxicity, with the risks of under oxygenation, e.g. hypoxaemia. The presence of poor gas exchange in the lungs will affect this balance, but the visualization of the decision might best be presented in relation to the preference toward the competing goals of over and under oxygenation rather than the physiological explanation of the cause of oxygenation problems.

Metaphor graphical representation have been applied in studies primarily to visualize diagnostic information concerning physiological function for the respiratory or cardiovascular system [20–24], in particular from the Westenskow group, with this having illustrated improved clinical performance in detecting critical events. Indeed, such visualizations, e.g. pictures of the lung annotated for mechanical properties are displayed on the screens of commercial ventilators. In contrast, little work has been performed on visualizing the preferences of decision makers when providing clinical decision support for mechanical ventilation. Such information is not intended to identify critical events, but rather to visualize the balances necessary when making clinical decisions, with the aim of visualizing the care delivered and the restoration of the patient to an optimal state [16].

This paper describes the application of metaphor graphics to represent clinical preferences related to mechanical ventilation. To do so, it describes the design of the Beacon Caresystem (Mermaid Care A/S, Nørresundby, Denmark), a commercial open loop CDSS for providing advice on the settings of mechanical ventilation based on physiological models and decision theory [25]. The paper describes the structure of the system, with focus on the use of decision theory and the graphical representation of clinical preference, with the physiological model structure having been described previously [11]. The design of this system presents a graphical representation of clinical preferences and their balance. It is postulated that this representation results in a number of graphical patterns which characterize situations where patients are, or are not, in optimal state. Here, optimal state is defined as the best balance between the competing goals of mechanical ventilation given the individual patient’s physiologic state. It should be noted that the optimal state defined here is not necessarily the best settings for the individual patient, but only the best given the specification of the physiologic and decision theoretic models used in the system. Identification of the graphical patterns may be important, as they may allow rapid evaluation of the need for, or lack of need for, changes in ventilator settings. To evaluate this hypothesis, i.e. that such patterns exist, the graphical presentations of patients taken from two previously conducted studies [13, 26] are analyzed retrospectively.

## Results

**Fig. 1:**
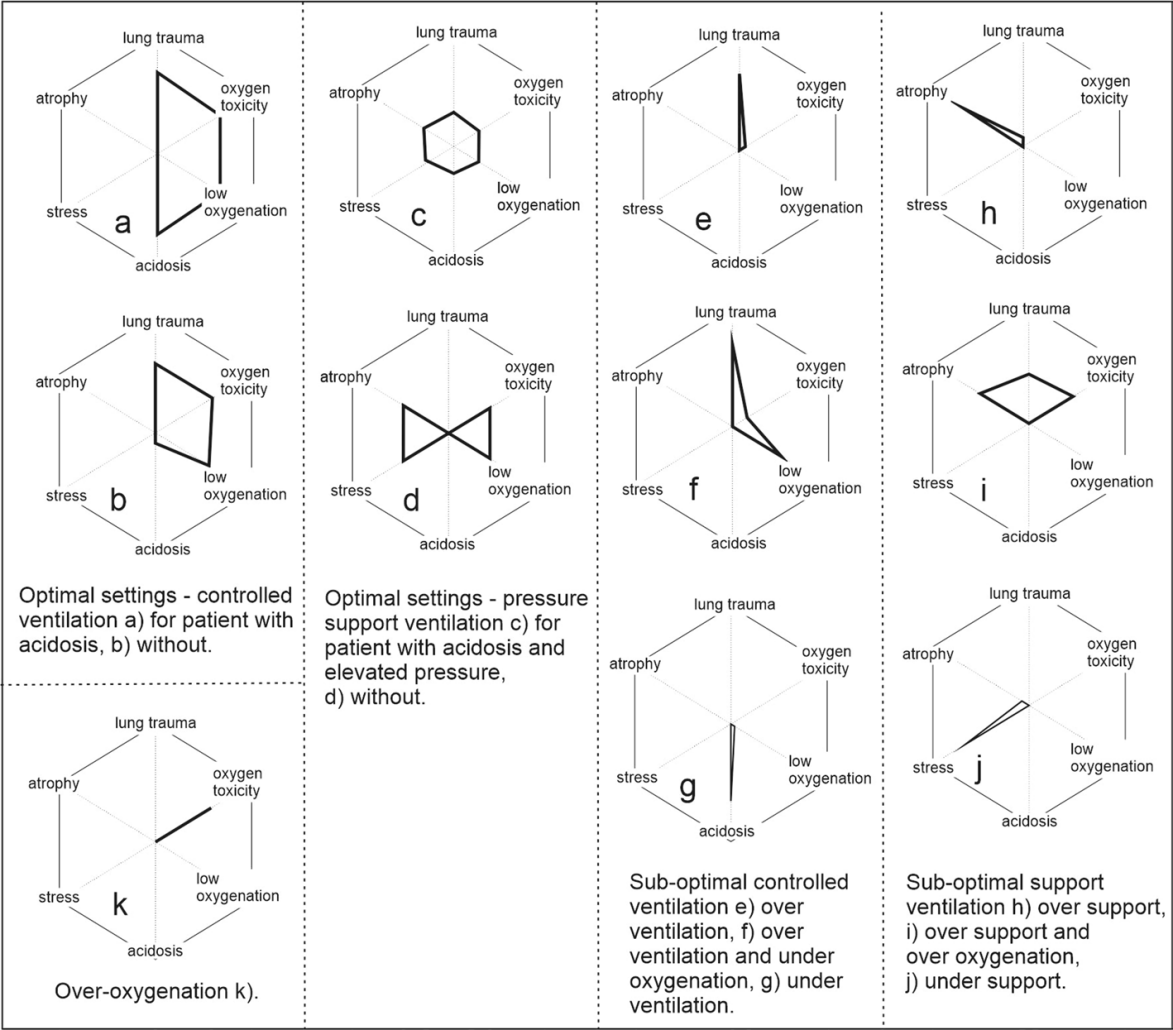
Patterns of clinical trade-offs identified in clinical data

Figure 1 illustrates the typical patterns of the clinical decision space seen in the studies. Optimally controlled patients in control mode ventilation (Fig. 1a, b) presented as a square or semi-circular picture on the right hand side of the hexagon (Additional file 1: Figs. S1–S3). Additional file 1: Figs. S1–S3 present such patterns for volume and pressure control modes in patients where adequate pressures, pH levels and oxygenation can be obtained. In contrast, Additional file 1: Fig. S3 illustrates a patient with extreme illness where the balance between pressure and pH level is achieved and hence optimal, but with severely abnormal values of both pressure and arterial pH, and a necessary use of permissive hypercapnia and hence low pH so as to reduce the dangerously high ventilator pressures.

Optimally controlled patients ventilated in pressure support presented either a circular pattern (Fig. 1c, Additional file 1: Figs. S4, S5), or as a bow tie (Fig. 1d, Additional file 1: Figs. S6, S7). These optimal patterns, could be seen at both low (Additional file 1: Figs. S4, S6) and moderate (Additional file 1: Figs. S5, S7), levels of support illustrating that the correct balance is individual patient specific. The yellow color of the hexagon on Additional file 1: Fig. S7 is due to the higher level of inspired oxygen and hence elevated risk of oxygen toxicity.

Sub-optimal control ventilation was seen for both volume and pressure control as over ventilation (Fig. 1e, f, Additional file 1: Figs. S8–S11), typically without oxygenation problems (Fig. 1e, Additional file 1: Figs. S8, S9) as an upwards spike; or with under oxygenation (Fig. 1f, Additional file 1: Figs. S10, S11) as spikes upward and to the lower right. Sub-optimal control ventilation was also seen as under ventilation (Fig. 1g, Additional file 1: Fig. S12) as a downward spike. The color coding in these examples reflects the severity of the spikes, with top pressures exceeding 25 cmH_2_O coded yellow, and the extremely low pH value (Additional file 1: Fig. S12) coded red. In these examples, advice was to counteract the direction of the spikes.

Sub-optimal pressure support ventilation was seen as over ventilation, as a left-upwards diagonal spike (Fig. 1h, Additional file 1: Figs. S13, S14). For patients with both over support, elevated pressure and over oxygenation, a quadrilateral presented above all three axes (Fig. 1i, Additional file 1: Figs. S15, S16), either at low (Additional file 1: Fig. S15) or high (Additional file 1: Fig. S16) pressure support (PS) levels. Additional file 1: Fig. S15 illustrates that simulated reduction in PS, from 8 to 6 cmH_2_O, results in a bow tie pattern, suggesting that 6 cmH_2_O is optimal for this patient.

Sub-optimal ventilation was also seen as under ventilation, as a left-downwards diagonal spike (Fig. 1j, Additional file 1: Figs. S17, S18) at both moderate (Additional file 1: Fig. S17) and high (Additional file 1: Fig. S18) pressure support levels. Additional file 1: Fig. S17 illustrates that simulated increase in PS, from 6 to 7 cmH_2_O, results in a predicted bow tie pattern, suggesting that 7 cmH_2_O is optimal for this patient.

Isolated patterns of over oxygenation present in both control and support mode ventilation as a right upward spike (Fig. 1k, Additional file 1: Figs. S19, S20). Under oxygenation without other sub-optimal settings was not observed in the data, but would present as a right downward spike.

## Discussion

This paper has presented a metaphor graphics approach to visualization of the decision space in mechanical ventilation. It has been shown that when designed using decision theory to represent the competing goals of mechanical ventilation, standard patterns emerge which visualize the decision space and hence the appropriateness of the current management strategy according to the CDSS.

Several patterns have been identified which visually represent the current compromises in the patient state and mechanical ventilation management. These include, semi-circular, round or bow tie patterns symbolizing optimal control, and then primarily spikes, in various directions, along with a rectangle above all axes for over support and over oxygenation in pressure support.

The most important part of this work is to highlight the known [16], but infrequently applied knowledge that presenting physiologic status and presenting the clinical decision space are not the same. There are currently few medical decision support algorithms or closed loop systems that adopt presentation of the decision space as a method to make transparent clinical goals and compromises. This paper illustrates that this is possible, that clear patterns appear, and provides numerous examples of these patterns from patient examples.

Recently, Shortliffe and Sepúlveda discussed the requirements for clinical decision support given the rapidly expanding volume of work in artificial intelligence (AI) [27]. They proposed six criteria which must be present if decision support systems are to be accepted and integrated into routine workflow. These are (1) Black boxes are unacceptable; (2) Time is a scarce resource; (3) Complexity and lack of usability thwart use; (4) Relevance and insight are essential; (5) Delivery of knowledge and information must be respectful; (6) Scientific foundation must be strong. These criteria present an excellent framework for discussing the potential benefit of the approach suggested in this paper.

When considering the approach taken here in relation to black-box technology, the approach can be considered in the context of the recent trend toward explainable artificial intelligence. Currently, AI is often used synonymously with machine learning techniques applied to big data. This has not always been the case, and historically AI was considered to include decision support tools based upon production rules or causal models and decision theoretic approaches [28]. When used synonymously for machine learning, the challenges of providing transparent reasoning are clear. Machine learning techniques generate associations, which are not necessarily understood from the inferred model structure. When applying models with causal reasoning, such as physiologic mathematical models, combined with decision theoretic models of preference, reasoning becomes transparent as illustrated by the examples included in this paper.

When considering the use of CDSS technology in relation to its requirements for clinical time, its complexity, and the relevance and appropriateness of the support, i.e. points 2–5 from Shortliffe and Sepúlveda, it is important to understand who the decision maker is, what is the nature of the decision and what is the experience of the decision maker. Deciding upon appropriate ventilator settings is a decision that can occur at several levels of expertise and in different conditions. Senior intensivists may discuss ventilator strategy for the patient at morning meetings or on ward rounds. Such discussion may involve deep physiological understanding as well as clinical trade-offs. In such a situation, detailed diagnostic information is likely required. At the bedside, decision making may be taken by the intensive care nurse, the respiratory therapist, or the junior doctor, dependent on the severity and complexity of the patient’s condition, and local and national norms. Here, depending on the expertise of the individual medical practitioner, diagnostic information may not be the major consideration, and visualization of clinical trade-offs may be a more appropriate level of complexity to integrate into clinical workflow. In addition, the ability to hide or expose the physiological measures at the corner of the hexagon allows the user to adjust the system to their own degree of expertise, providing them with a transparent link from a symbolic to a physiologic representation and a varying level of complexity. Transparency might be further enhanced by the simulation function. This is unlikely to be used by the busy bedside practitioner, but may allow for bedside teaching on ward rounds exploring the different options for the patient. Given the increasing role of high-fidelity mannequin-based training of critical incidences [29, 30], it would seem appropriate to incorporate bedside technology allowing exploration and discussion of transparent clinical decisions in a teaching setting. The most detailed diagnostic information—shunt fractions, blood parameters and respiratory drive—might only be viewed by the most experienced clinicians.

A flexible structure providing illustration of the clinical decision space and diagnostic information, available through causal models and decision theory, allowing the user to choose the level of detail they view, may, therefore, be an ideal approach to provide relevant complexity. It may reflect the time constraints and expertise of the individual user, and ensure transparency and prevent black-box systems. In addition, the physiologic approach may ensure scientific rigor, as physiological models at the organ level are well founded and can be tuned to represent the individual patient [11]. Preference functions are, by their nature, subjective. However, displaying the effects of these as compromises may promote rational clinical reasoning, acknowledge different opinions and allow for debate and potentially modification of preference functions. Such functionality would be important if a learning culture is to be promoted [31].

This paper has presented a series of patterns from clinical examples in previously conducted studies. It has not been the intention of this paper to report the outcomes of using the system, which have been described previously [13, 30]. While these patterns appear intuitive, and are used to illustrate the more general point that presentation of the decision space may be illustrative, this paper has not evaluated the acceptability of the patterns presented here or evaluated their implications on clinical decision making or workflow. In this context, comparing the ease and speed of interpretation for standard data presentation compared with displays illustrating the decision space might be useful. Indeed, this has been applied previously when comparing standard data presentation with data describing physiological interpretation [20]. Randomized controlled trials are underway evaluating the clinical efficacy of the advice provided by the decision support system presented here [32], but it is clear that routine use of such a system will not depend on efficacy alone [27], and evaluation of the usability of the system is important if this, and other such systems, are to be integrated into routine clinical workflow.

## Conclusions

This study illustrates that visual patterns can be identified which describe the trade-offs required in mechanical ventilation. These patterns may have potential to reduce information overload, and help in simple and rapid identification of sub-optimal settings.

## Methods

### CDSS description

**Fig. 2:**
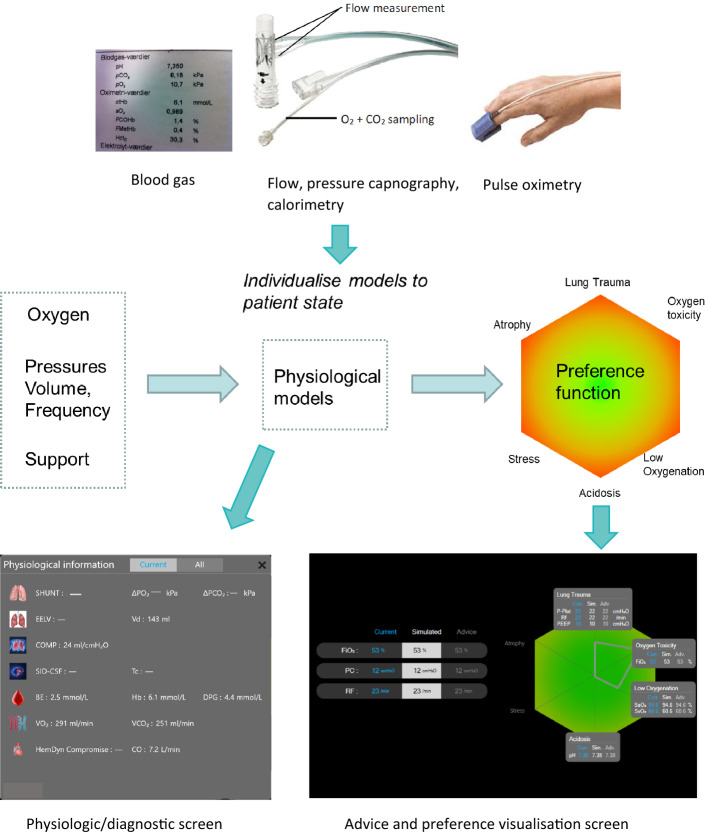
The structure of the Beacon Caresystem and output screens illustrating physiologic state and advice/preference visualisation screen illustrating a patient in control mode ventilation. The following abbreviations are used on the physiologic screen (all others in text): Shunt: pulmonary shunt; ΔPO_2_: partial pressure O_2_ drop due to low V/Q; ΔPCO_2_: partial pressure CO_2_ drop due to high V/Q; EELV: end expiratory lung volume; Vd: serial dead space; COMP: respiratory system compliance; SID-CSF: model estimated strong ion difference of the CSF; Tc: Threshold to central respiratory drive; BE: base excess; Hb: haemoglobin; DPG: 2,3 diphosphoglycerate; VO_2_: oxygen consumption; VCO_2_: carbon dioxide production; CO: cardiac output. For the physiologic screen where values are missing, they were not necessary for this specific patient and not estimated by the system

Figure 2 illustrates the structure of the Beacon Caresystem and two of the CDSS’s screen outputs. The system provides advice on changes in mechanical ventilator settings based upon mathematical physiological models and mathematical models of clinical preference. The system functions by individualizing the mathematical physiological models to the patient’s state by tuning physiologic model parameters to fit measurements taken by the system. These measurements come from built in sensors measuring flow, pressure, volumetric capnography, indirect calorimetry and pulse oximetry; as well as user input blood gas values. The system connects to the ventilator to obtain information about ventilator settings and ventilation mode. These mathematical models and their tuning have been described in detail previously [11].

**Fig. 3:**
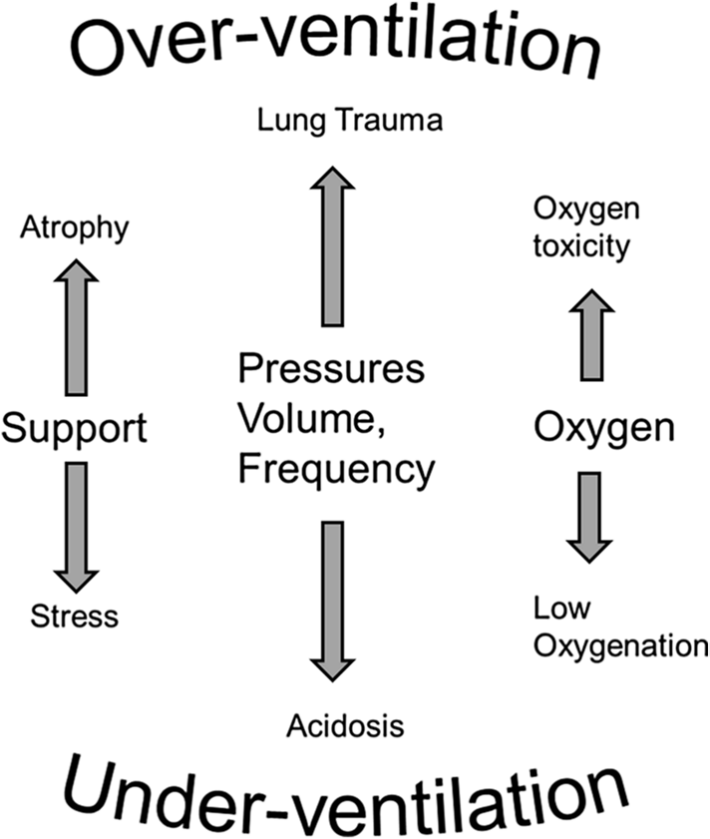
Balances necessary when setting the appropriate levels of mechanical ventilation

Following tuning of the physiologic models, the system then calculates patient specific predictions of changes in ventilation and calculates the preference values associated with competing goals. These competing goals are described in detail below in relation to Fig. 3, but are represented as quantitative values of penalty associated with each of the detrimental effects of mechanical ventilation shown on the axes of a hexagon. The net detrimental effect of any strategy is then the total, unweighted, sum of the penalties for each of the individual detrimental effects in a decision theoretic approach [12]. Searching through possible combinations of ventilator settings then allows identification of the settings with the least total penalty, i.e. the optimal settings. As optimal settings may be substantially different from current settings, advice is then generated to take a step toward these. Following change of ventilator settings by the clinician, a period of 5–20 min is waited to ensure the full effects of the ventilator changes are complete, and new advice then generated. If the patient is in the optimal state, described by a minimum total penalty, then no further advice is generated until the patient state changes. In this way, the frequency of advice depends upon the stability of the patient and the difference between current and optimal ventilator settings. The screens illustrated in the Additional file 1 of this manuscript each present a single piece of advice, as such they reflect a step change in ventilation toward the goal. For each step, the maximum change advised upon for each setting is: for inspired oxygen 5%; for pressures 2 cmH_2_O (3 cmH_2_O for advice to increase positive end expiratory pressure (PEEP) if the value of PEEP is less than 10 cmH_2_O); for tidal volume 50 ml and for respiratory frequency 3 breaths/min. As noted above, these individual advice steps usually occur to move the patient toward an optimal state. If, for example, the system believes the optimal pressure support to be 5 cmH_2_O lower than the current value, then this will be presented as three steps of reduction in levels of 2, 2 and 1 cmH_2_O over a period of about 30 min. Following each change, the system will learn from the patient’s response to that change, modifying therapy if necessary.

The visualization of the patient state is performed using three different screens, two of which are illustrated in Fig. 2. The first is a diagnostic screen which illustrates the values of model parameters describing each of the relevant physiological systems, analogous to those described in the introduction. This is not considered further in this paper. The second is the screen visualizing advice, and the hexagon representation of clinical preference. The third screen is a history screen, presenting the progression of the patient as multiple hexagon displays. The hexagon displayed on the second screen, is designed to reflect the competing goals of mechanical ventilation, as illustrated in Fig. 3 where each vertical axis of the hexagon represents a competing goal of mechanical ventilation. Figure 3 illustrates the competing goals when adjusting pressure support; pressures, volume or frequency in control mode ventilation; or inspired oxygen. For oxygen, too little is associated with low oxygenation or hypoxia, too much is associated with oxygen toxicity [33]. For pressures, volume or frequency in control mode ventilation, too little of these is associated with reduced levels of minute ventilation and carbon dioxide elimination, and hence blood acidosis. Too high settings of volume, pressure or frequency are associated with lung trauma due to ventilator-induced lung injury [1]. Patients with spontaneous breathing activity are often ventilated in pressure support mode. Too much pressure support reduces the patient’s drive to breathe, resulting in lower respiratory frequency and respiratory muscle effort and consequently risk of respiratory muscle atrophy [34]. Too little pressure support can result in stressful breathing patterns due to inappropriate demands on the patient’s respiratory muscles or cardiovascular system. These six conflicting goals, illustrated in Fig. 3, are the axes of the hexagon presented in Fig. 2. Each axis is associated with a mathematical preference function which assigns penalty to physiological variables describing the risks associated with each axis. These variables are as follows: for oxygen toxicity, penalty is assigned to elevated levels of inspired oxygen fraction; for low oxygenation, penalty is assigned to a combination of simulated arterial and mixed venous oxygen saturation, the latter representing systematic deprivation of oxygenation in relation to tissue utilization. For lung trauma, penalty is assigned to a combination of tidal volume, driving pressure and respiratory frequency, i.e. in line with current thinking that increase in any of these increase the mechanical power of breathing and hence the potential lung damage [35]. For acidosis, penalty is assigned to the simulated arterial pH. For the risk of respiratory muscle atrophy, penalty is associated with low levels of respiratory frequency, with modification made for high values of tidal volume in situations where it is clear that respiratory muscles are active. For the risk of stress due to low levels of pressure support, penalty is assigned to the ratio of respiratory frequency to tidal volume, adjusted to predicted body weight, as described previously [36].

The hexagon, the symbols drawn on it, and the whole of the advice and preference visualization screen can be interpreted with reference to Fig. 2, or to the many patient examples included in the Additional file 1 for this manuscript, as follows. Advice screens (Additional file 1: Figs. S1–S20), are separated into a left and right hand side. The left hand side gives values of the current ventilator settings (in blue), and those advised (in grey), along with simulated values (in black). Simulated values allow the user to click on these fields and simulate the changes in physiological variable and associated penalties for any ventilator settings. The setting variables shown on the left hand side depend on the ventilator mode, and the ventilator settings for which advice is available. The right hand side of the screen represents the hexagon description of the balances necessary to make decisions. Drawn on the hexagon are two symbols in blue and grey, respectively. The blue symbol is that which represents the penalties for the current ventilator settings. The grey is that representing advice, or when the simulation function of the left hand side is activated, represents the user simulated preferences for any ventilator settings. The blue and grey symbols are drawn by joining the points representing the different penalty values for each of the competing goals. For patients in control mode ventilation without spontaneous breathing activity, the symbol fills only the right hand side of the hexagon, as values related to respiratory muscle atrophy or respiratory stress are not shown for patients who do not have respiratory muscle activity (Additional file 1: Figs. S1–S3, S8–S12). For patients with spontaneous breathing activity, the symbol is drawn to join the penalty values across all axes on the hexagon (Additional file 1: Figs. S4–S7, S13–S20). Three colors are used for the background of the hexagon (green, yellow, red) with red symbolizing situation where penalty is high, yellow medium, and green low on at least one of the axes. Axes are rescaled dependent upon the highest penalty, so that a smaller area shown on the red part of a hexagon can be a higher penalty than a larger area on hexagon where axes have been rescaled to only show only the green area. In addition to the symbols, the physiologic values of variables describing each preference can be displayed by touching the corners of each hexagon axis. The values associated with current, simulated and advised ventilator settings are shown for each variable on the respective axis. These are shown for all figures in Additional file 1, but will not be at the bedside unless the user activates them.

### Data and analysis

Data from 95 patients from previously published studies [13, 26] were retrospectively analyzed. Study [13] applied the advice of the Beacon Caresystem to 72 patients ventilated in control and support modes over 4–8 h. Study [26] studied 23 patients in pressure support mode, and investigated the advice following interventions to over and under support the patients by modifying pressure support levels.

In this study, the hexagons representing each piece of advice from the previous two studies were analyzed visually. In doing so, it was investigated whether there were repeated patterns of shapes drawn on the hexagons and whether these represented specific clinical scenarios. It was assessed whether it was possible to tabulate these examples so as to provide a clinical understanding of these repeated patterns, supported by numerous patient examples of the nature of these patterns. Where patterns were seen frequently, two examples were included in Additional file 1, where these illustrated the same pattern at different conditions of ventilator mode and level. This strategy was taken to illustrate as many different patterns as possible without an overwhelming number of clinical examples.

## Supplementary Information

**Additional file 1.** Supp. Mat: Transparent decision support for mechanical ventilation.

## Data Availability

The screen displays included in Additional file 1 represent the data for this manuscript.

## Abbreviations

ICU: Intensive care unit
CDSS: Clinical decision support systems
ESM: Electronic supplementary material
PS: Pressure support
AI: Artificial intelligence
PEEP: Positive end expiratory pressure
Shunt: Pulmonary shunt
ΔPO_2_: Partial pressure O_2_ drop due to low V/Q
ΔPCO_2_: Partial pressure CO_2_ drop due to high V/Q
EELV: End expiratory lung volume
Vd: Serial dead space
COMP: Respiratory system compliance
SID-CSF: Model estimated strong ion difference of the CSF
Tc: Threshold to central respiratory drive
BE: Base excess
Hb: Haemoglobin concentration
DPG: 2,3 Diphosphoglycerate
VO_2_: Oxygen consumption
VCO_2_: Carbon dioxide production
CO: Cardiac output
