# Supplementary material for: Transparent decision support for mechanical ventilation using visualization of clinical preferences
Source: Biomed Eng Online. 2022 Jan 24;21:5. doi: 10.1186/s12938-021-00974-5 (PMC8785460; doi:10.1186/s12938-021-00974-5)
Supplement: Supplementary file 1 — Additional file 1. Supp. Mat: Transparent decision support for mechanical ventilation. [file 12938_2021_974_MOESM1_ESM.docx]

Supplementary material for:

Transparent decision support for mechanical ventilation using visualization of clinical preferences.

Stephen Edward Rees^1^, Savino Spadaro^2^, Francesca Dalla Corte^3^, Nilanjan Dey^4^, Jakob Bredal Brohus^5^, Gaetano Scaramuzzo^2^, David Lodahl^6^, Robert Ravnholt Winding^4^, Carlo Alberto Volta^2^, Dan Stieper Karbing^1^.

1 - Respiratory and Critical Care Group, Department of Health Science and Technology, Aalborg University, Aalborg, Denmark.

2 - Department of Morphology, Experimental Medicine and Surgery, Section of Anaesthesia and Intensive Care, Arcispedale Sant’ Anna, University of Ferrara, Ferrara, Italy.

3 -  Department of Anaesthesia and Intensive Care, Humanitas Clinical and Research Center-IRCCS, Rozzano, MI, Italy.

4 - Department of Anesthesia and Intensive Care, Regions Hospital Herning, Denmark.

5- Mermaid Care A/S, Nørresundby, Denmark.

6 - Department of Anesthesia and Intensive Care, Aalborg University Hospital, Aalborg University, Aalborg.

**Introduction**

This supplementary material illustrates patient cases of ventilator settings and advice, hexagon patterns, and the values of variables used in calculation of clinical preference on the edges of the hexagon. Standard patterns are presented for the numerous clinical situations described in the main text and table 3.

Please note: some text may be small when printing these case, particularly for text in blue. In this case we recommend you view them on the screen and zoom in to the relevant values which then should be of sufficient resolution.

**Optimal settings in control mode ventilation**


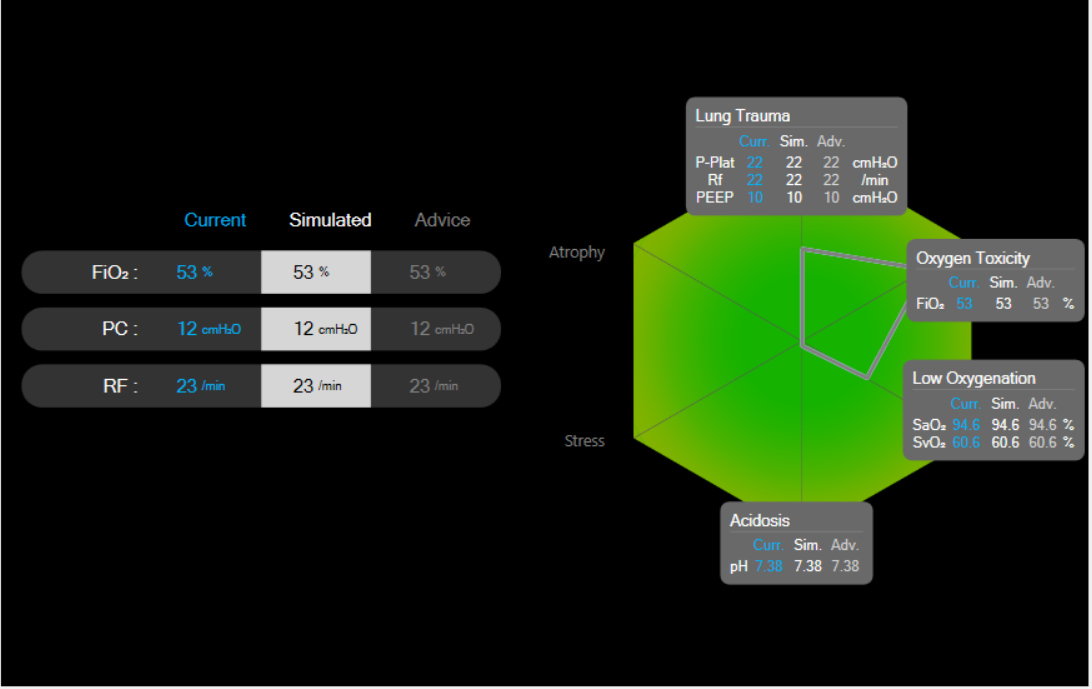


Figure A1 – Patient in pressure controlled mode, advice to maintain settings.


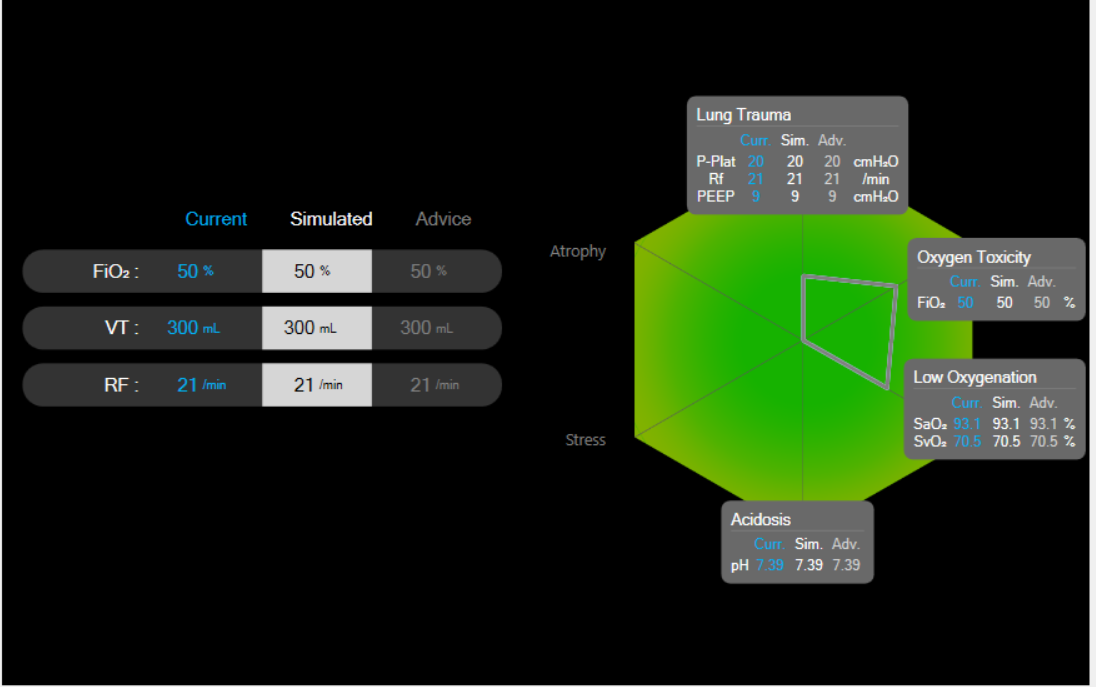


Figure A2 – Patient in volume controlled mode, advice to maintain settings.


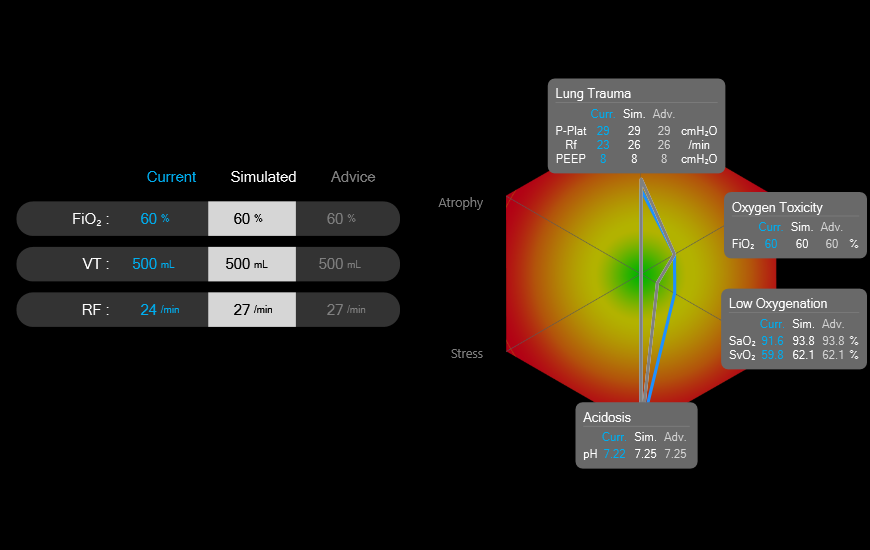


Figure A3 – Patient in volume controlled mode, a red hexagon with both high risk of lung trauma and acidosis. A good balance despite the severity of patient condition with advice to slightly increase respiratory frequency for marginal correction of acidosis (predicted oxygenation improves due to Bohr-Haldane effect).

**Optimal settings in pressure support ventilation**


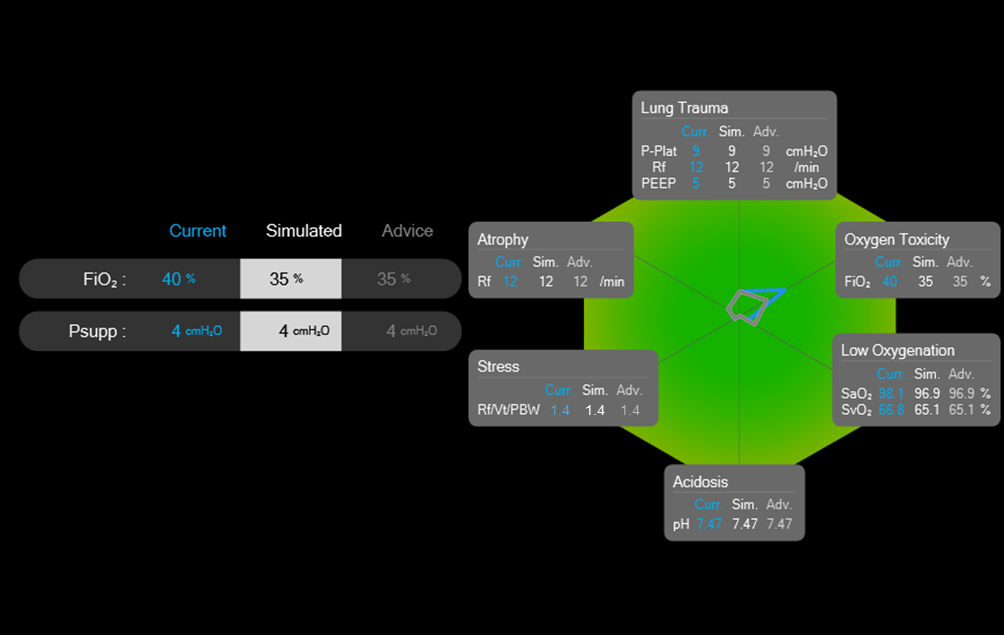


Figure A4 – Patient in pressure support mode with low PS levels, advice to maintain PS settings, with a reduction in inspired oxygen.


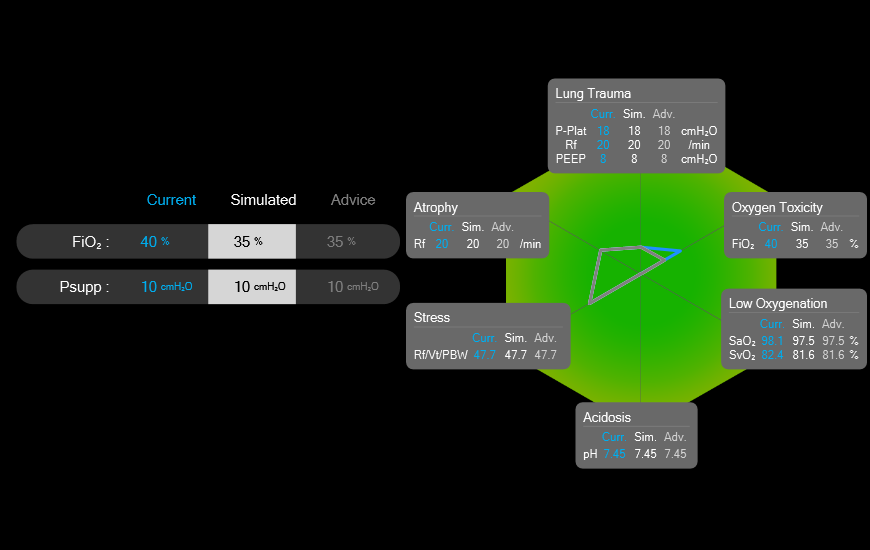


Figure A5 – Patient in pressure support mode with moderate PS levels, advice to maintain PS settings, with a reduction in inspired oxygen.


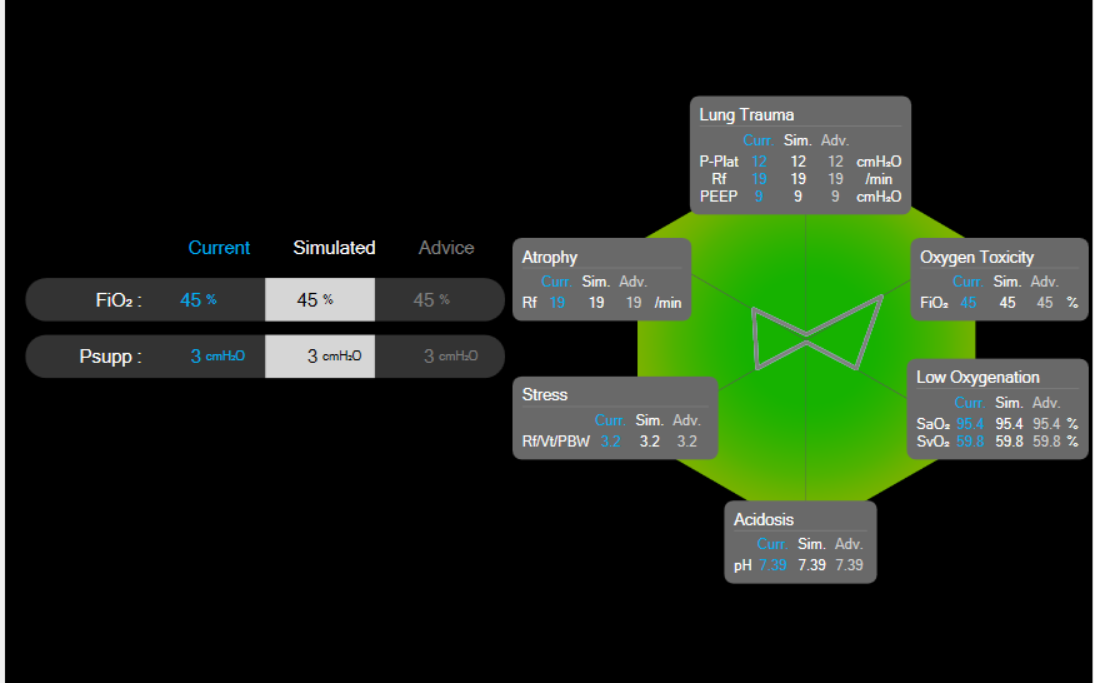


Figure A6 – Patient in pressure support mode with very low PS level, advice to maintain settings.


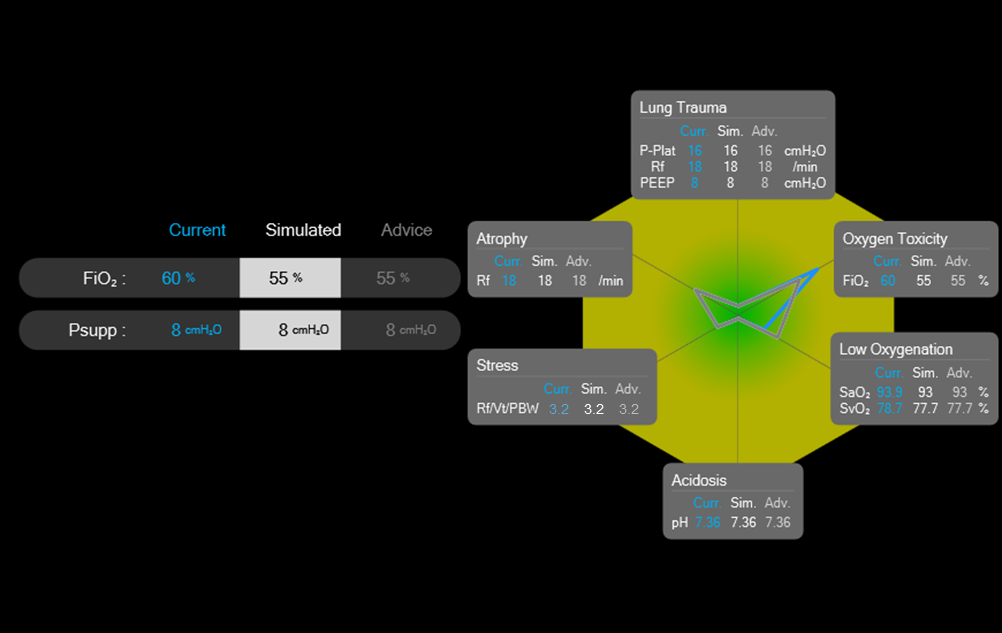


Figure A7 – Patient in pressure support mode with moderate PS levels, advice to maintain PS settings, with a reduction in inspired oxygen.

**Over ventilated in control mode ventilation**


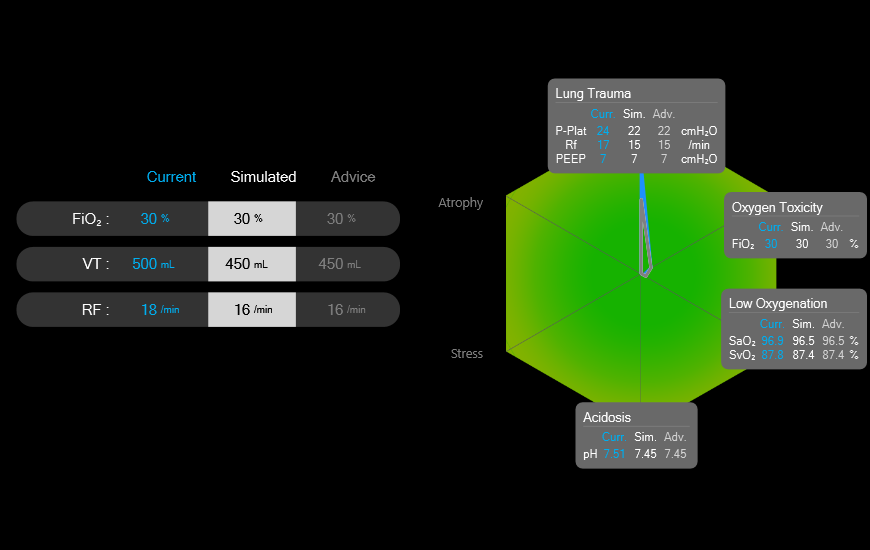


Figure A8 - Patient in volume control mode, a ‘spike’ toward lung trauma, and advice to reduce tidal volume and frequency, also allowing for a reduced alkalosis. Green hexagon as top pressure level does not exceed 25 cmH_2_O.

.


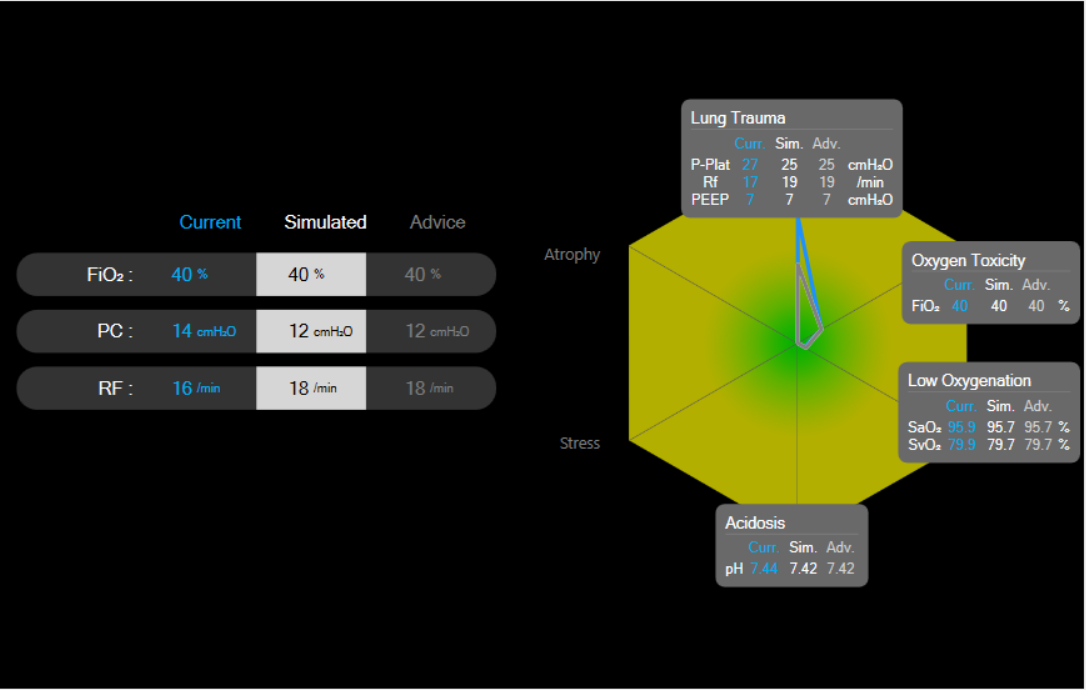


Figure A9 - Patient in pressure control mode, a ‘spike’ toward lung trauma, and advice to reduce delivered pressure and frequency, also allowing for a reduced alkalosis. Yellow hexagon as top pressure level exceeds 25 cmH_2_O.

**Over ventilated combined with under oxygenation in control mode ventilation**


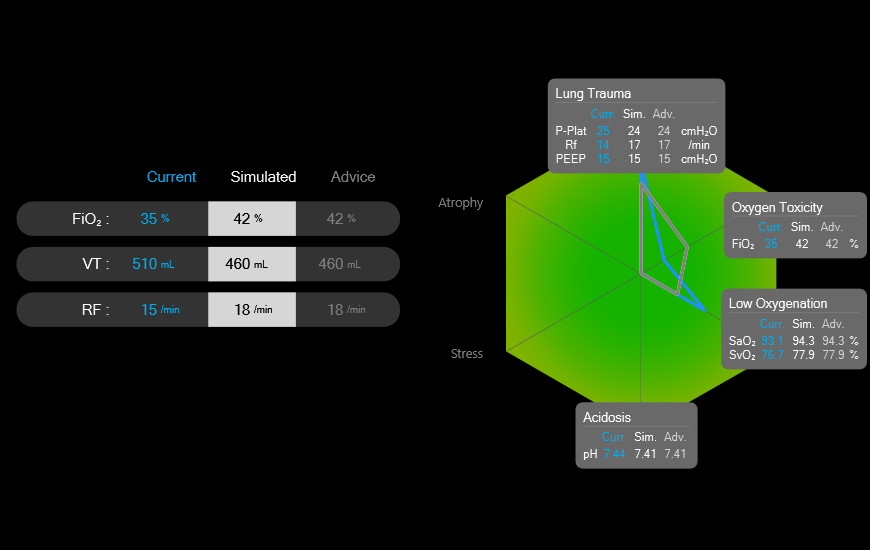


Figure A10 – Patient in volume control mode, a ‘spike’ toward lung trauma and low oxygenation. Advice to reduce tidal volume, compensated by increased frequency to maintain pH, and increased inspired oxygen.


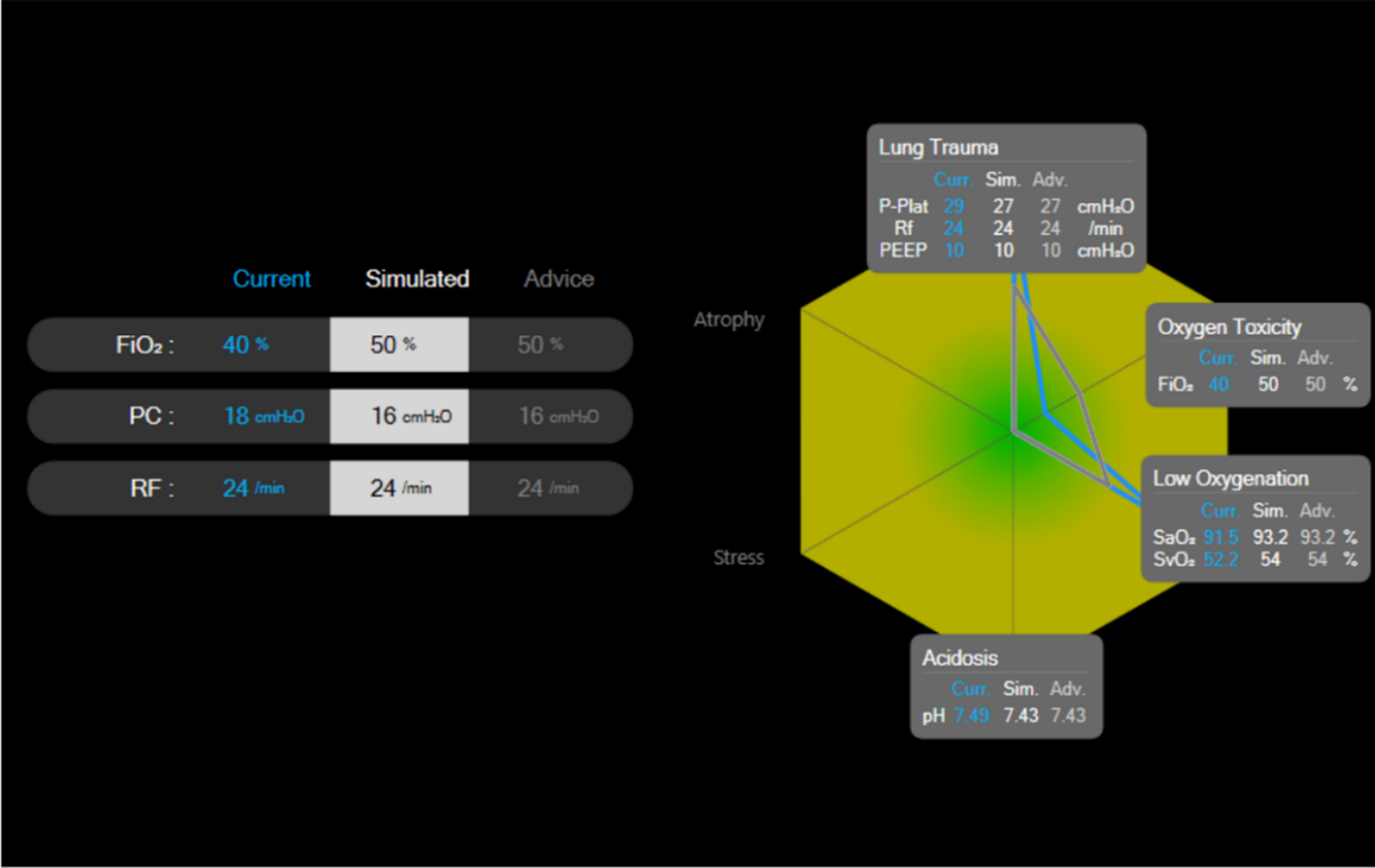


Figure A11 – Patient in pressure control mode, a ‘spike’ toward lung trauma and low oxygenation. Advice to reduce pressure and increased inspired oxygen.

.

**Under ventilated in control mode ventilation**


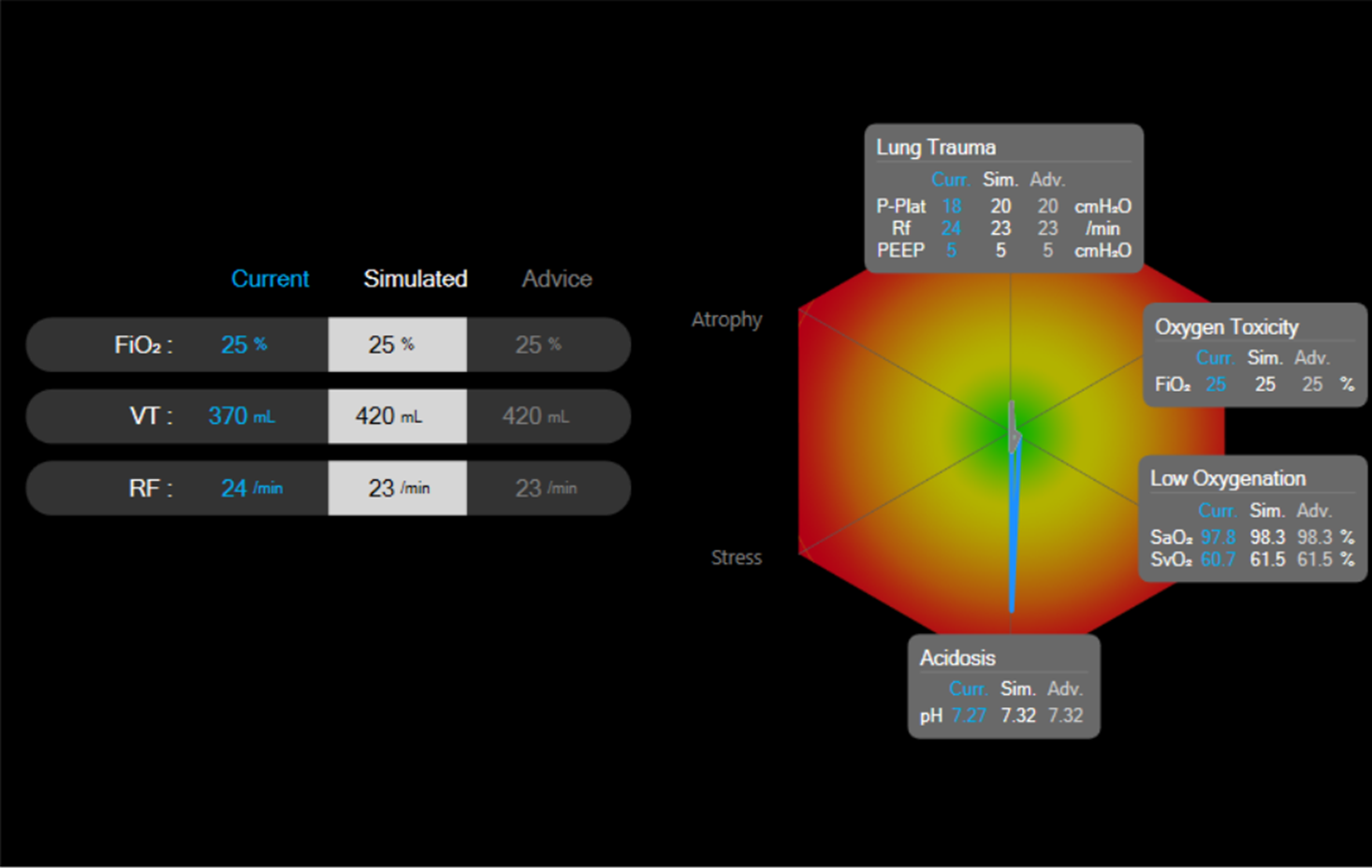


Figure A12 – Patient in volume control mode, a ‘spike’ toward acidosis and advice to increase tidal volume, with little increased risk of lung trauma.

.

**Over support in pressure support ventilation**


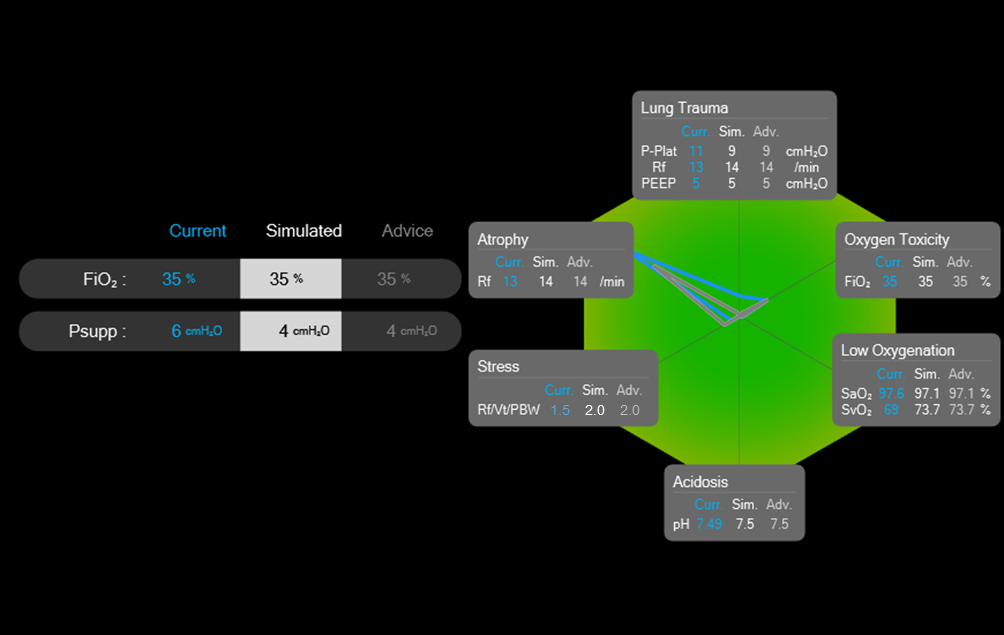


Figure A13 – Patient in pressure support mode, a ‘spike’ toward respiratory muscle atrophy and advice to decrease pressure support with little elevated risk of patient stress.

.


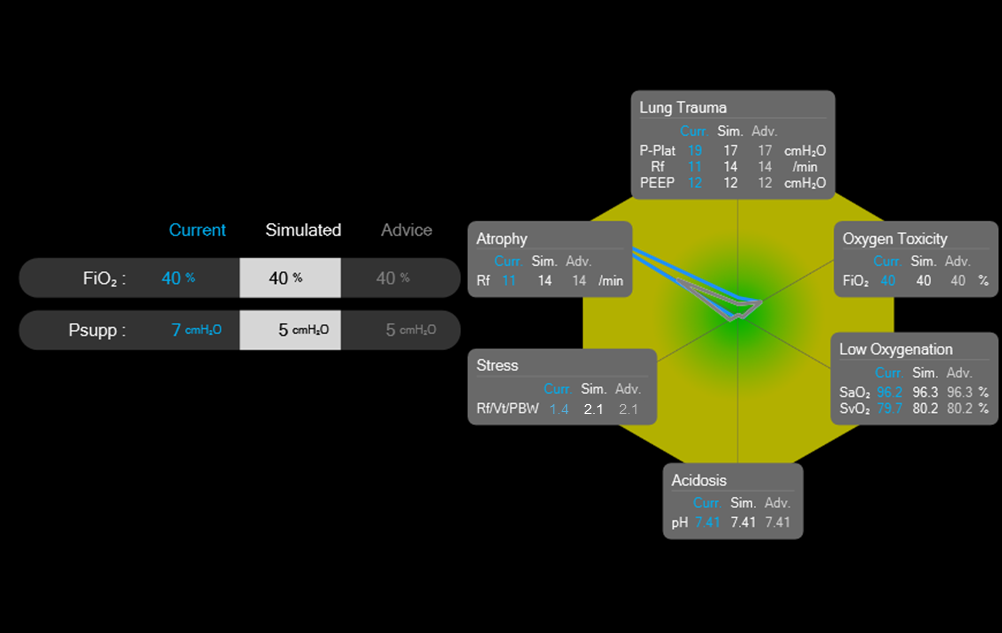


Figure A14 – Patient in pressure support mode, a ‘spike’ toward respiratory muscle atrophy and advice to decrease pressure support with little elevated risk of patient stress. Yellow hexagon due to low levels of respiratory rate.

**Over support and oxygenation pressure support ventilation**

**
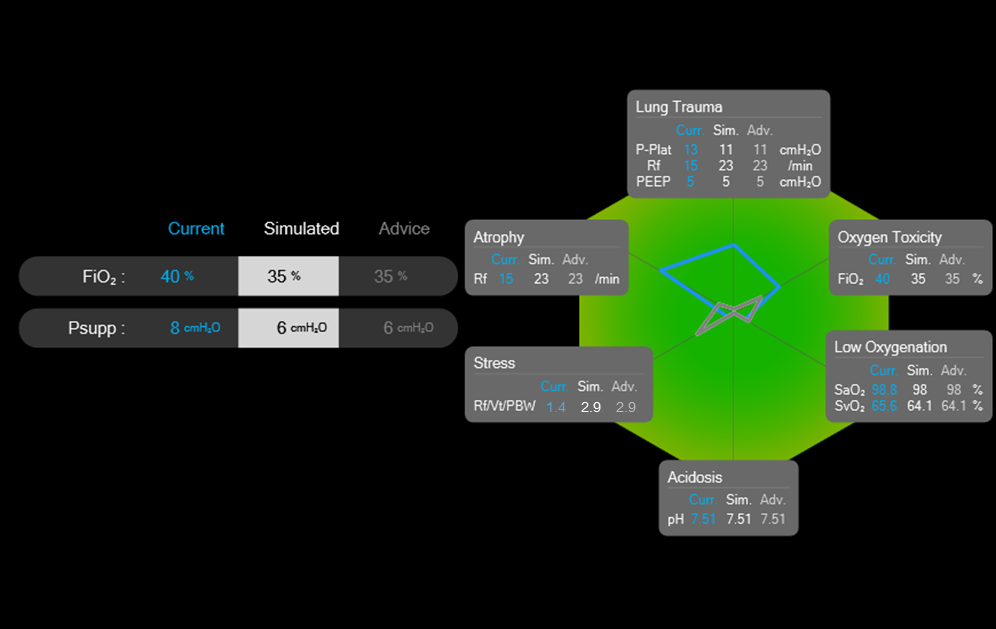
**

Figure A15 – Patient in pressure support mode with the blue symbol elevated toward atrophy, lung trauma and oxygen toxicity, advice to decrease pressure support and oxygen level, note the prediction that this will result in a “bow tie” pattern as shown by the grey symbol. Moderate to low PS levels.


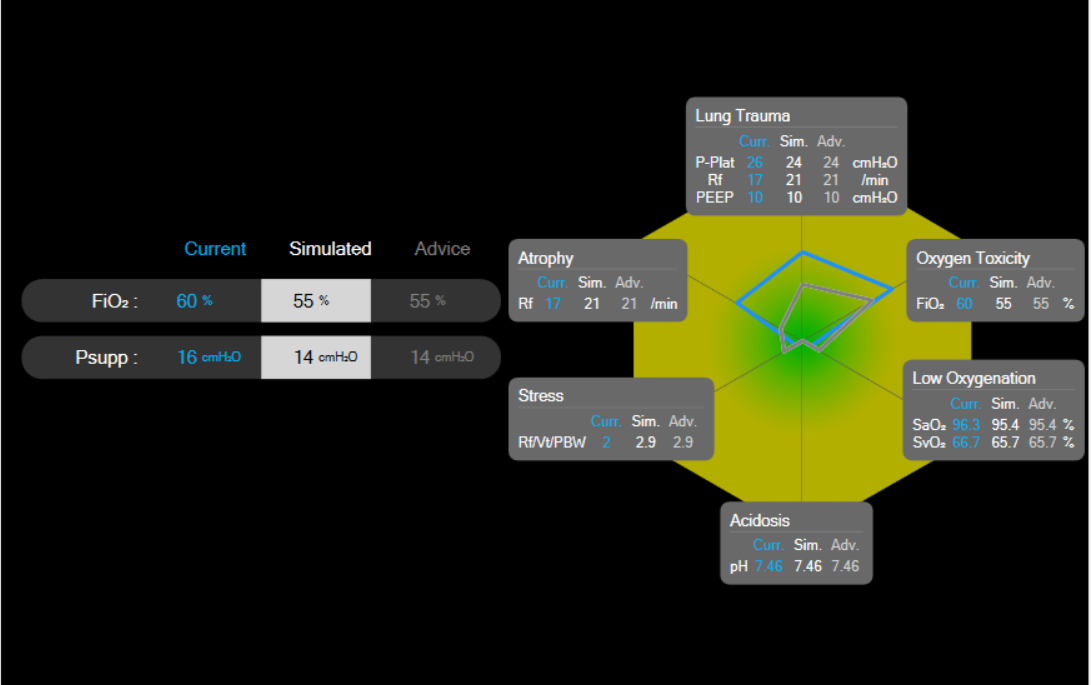


Figure A16 – Patient in pressure support mode with the blue symbol elevated toward atrophy, lung trauma and oxygen toxicity, advice to decrease pressure support and oxygen level. High PS levels.

**Under support in pressure support ventilation.**

**
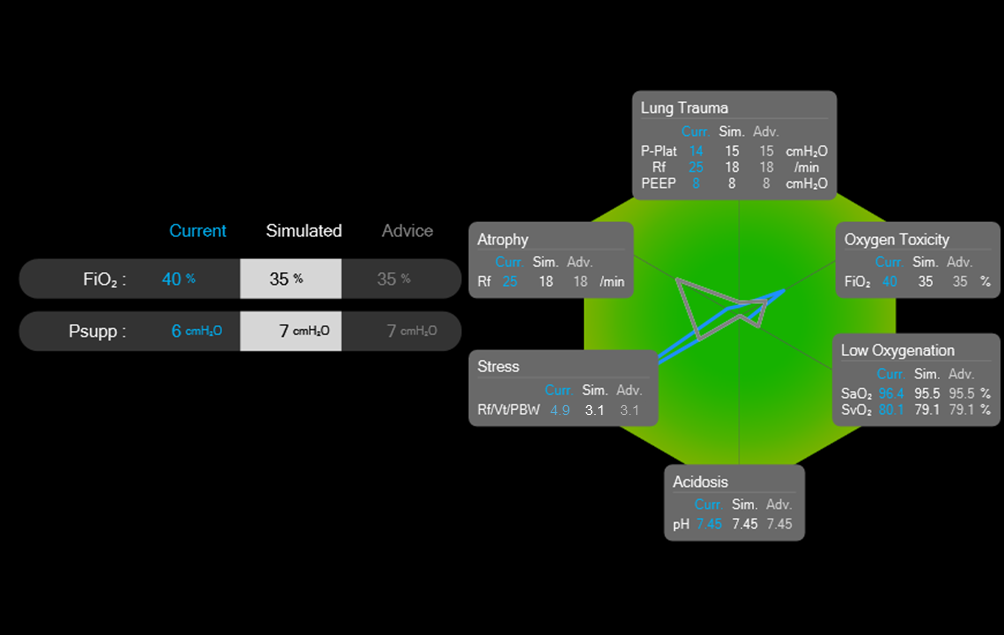
**

Figure A17 – Patient in pressure support mode with a ‘spike’ toward respiratory stress and advice to increase pressure support. Note prediction is that this will result in a “bow tie” pattern as shown by the grey symbol. Low PS levels.


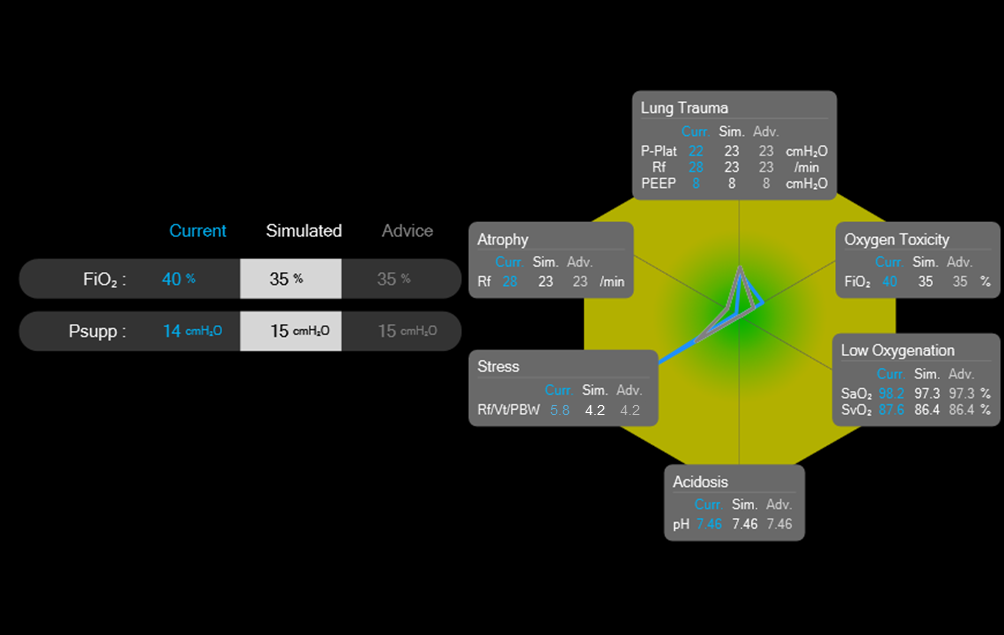


Figure A18 – Patient in pressure support mode with a ‘spike’ toward respiratory stress and advice to increase pressure support. High PS levels.

**Over oxygenation alone**


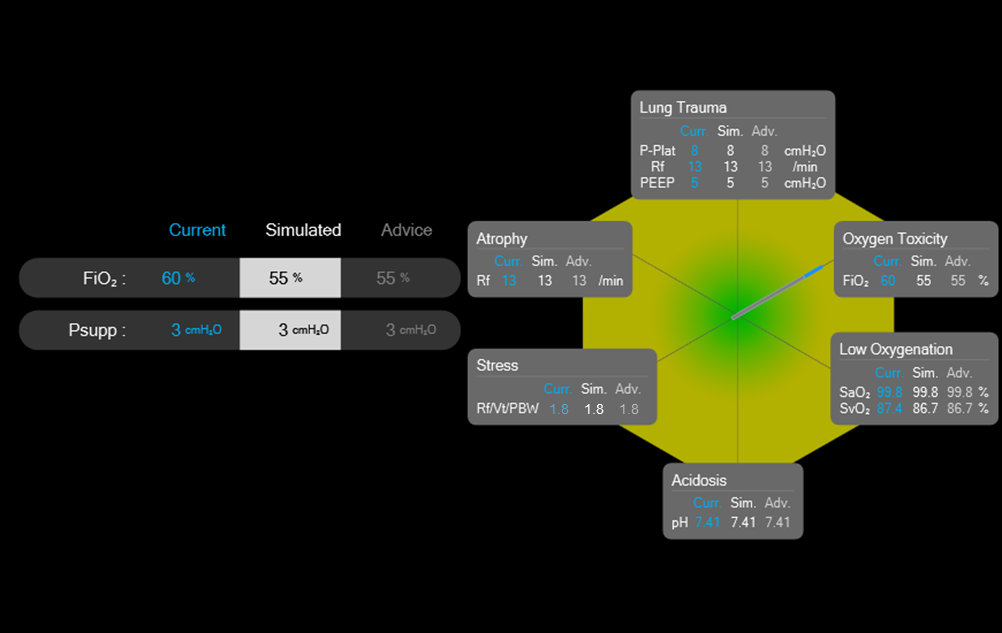


Figure A19 – Patient in pressure support mode with low support levels, a ‘spike’ toward oxygen toxicity and advice to decrease inspired oxygen.


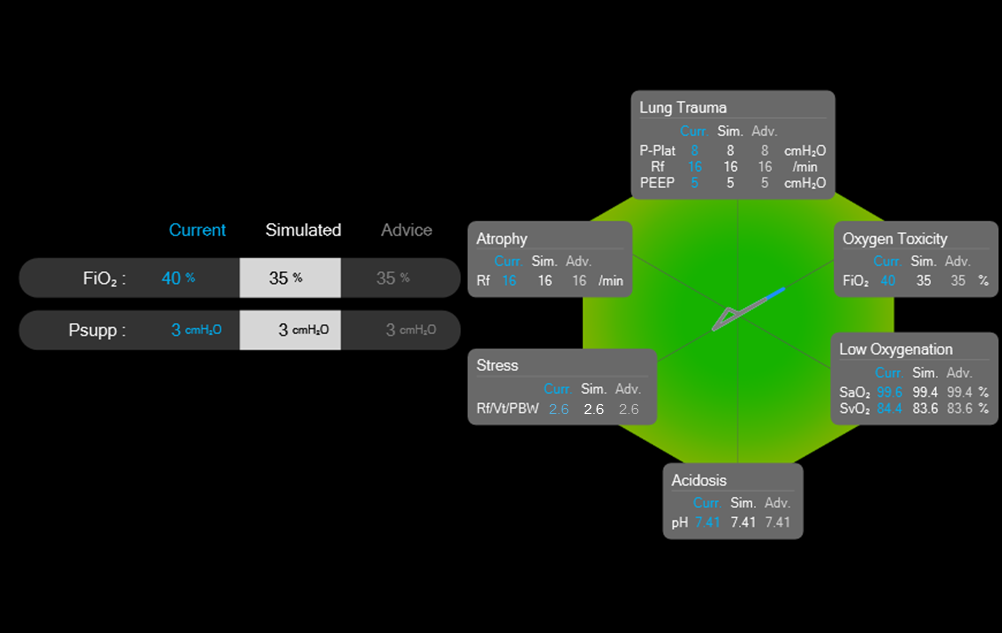


Figure A20 – Patient in pressure support mode with low support levels, a ‘spike’ toward oxygen toxicity and advice to decrease inspired oxygen.
